# Association of co-occurring mental health problems with hepatitis C status among young people who inject drugs in rural New Mexico, 2016–2018

**DOI:** 10.1186/s13722-022-00340-3

**Published:** 2022-10-20

**Authors:** Akash Gupta, Fatma M. Shebl, Yao Tong, Katherine Wagner, Ingrid V. Bassett, Kimberly Page, Erin L. Winstanley

**Affiliations:** 1 Medical Practice Evaluation Center, Massachusetts General Hospital, 100 Cambridge St, 16th Floor, Boston, MA 02114 USA; 2 University of New Mexico, Albuquerque, NM USA; 3 West Virginia University, Morgantown, WV USA

**Keywords:** Stress disorders, Post-traumatic, Injection drug use, Hepatitis C, Mental health, Opioid use disorder treatment

## Abstract

**Background:**

Injection drug use (IDU) remains the strongest risk factor for hepatitis C virus (HCV) in the United States. HCV rates are increasing in rural areas among young adult people who inject drugs (PWID). People with HCV and PWID have disproportionate rates of mental health problems; however, it is unclear whether risky injection behaviors mediate the association between mental health problems and HCV. We examined the association between mental health problems and HCV in a rural cohort of young adult PWID, with the goal of informing rural service delivery.

**Methods:**

We conducted a secondary analysis of cross-sectional data from a convenience sample of young adult PWID in 2 rural counties in New Mexico. Participants were recruited from 2 community venues between September 2016 and May 2018. Associations between mental health problems and HCV were examined using bivariate (Fisher’s exact) and multivariable modified Poisson regression analyses (with robust standard errors). Using structural equation modeling (SEM), we assessed duration of IDU and receptive syringe sharing (RSS) as mediators of this relationship.

**Results:**

A total of 263 patients were enrolled, with a median age of 26.1 years. The majority were male (66.3%) and Hispanic/Latino (a) (87.6%). The median age first injected was 19 years, and over half reported having ever engaged in RSS (53.4%). At least one mental health problem was reported by 60.1% of participants, with post-traumatic stress disorder (PTSD) being the most prevalent condition (42.2%). A majority (60.9%) tested positive for HCV antibody, and just under half (45.7%) of all participants tested positive for HCV ribonucleic acid. In SEM, PTSD had a significant total effect on HCV (τ = 0.230, P = 0.05), and this relationship was partially mediated by duration of IDU (αβ = 0.077, P = 0.03). The association between mental health problems and HCV was partially mediated by duration of IDU and the sequential mediation of duration of IDU and RSS (αβ + αββ = 0.091, P = 0.05).

**Conclusions:**

High HCV rates among young adult PWID in rural New Mexico may be partly explained by mental health problems, duration of IDU and RSS. Mental health services for young adult PWID in rural areas may help decrease HCV transmission in rural areas.

*Trial Registration* N/A.

**Supplementary Information:**

The online version contains supplementary material available at 10.1186/s13722-022-00340-3.

## Background

Injection drug use (IDU) remains the strongest risk factor for acquisition of hepatitis C virus (HCV) in the United States (US) [1, 2]. Over the past decade, HCV incidence has increased among younger people who inject drugs (PWID) in both urban and rural areas of the US [1, 3, 4]. An analysis of national surveillance data showed a 13% annual increase in HCV incidence in young persons in non-urban counties, compared to 5% annually in urban counties [3]. The Centers for Disease Control & Prevention performed an analysis of counties vulnerable to HIV and HCV infections among PWID, and found that the counties were overwhelmingly rural [5]. Among PWID, certain risk factors for HCV acquisition are well-established, including frequency of injection, duration of IDU, and sharing of injecting equipment, including receptive syringe sharing (RSS) and sharing previously used ancillary equipment [6–9].

Mental health problems have been demonstrated to be over-represented among young PWID [10]. Notably, the prevalence of adverse childhood events is up to 80% in patients seeking treatment for substance use disorders (SUDs), and rates of post-traumatic stress disorder (PTSD) can be as high as 30–60% [11, 12]. There is less research available focusing on mental health problems and PTSD among people with SUDs in rural settings. Still, one cohort of patients receiving buprenorphine treatment in West Virginia found high (> 4 categories) adverse childhood events scores in 54.3% of patients, with a higher proportion among females compared to males [13].

Additionally, research suggests that a history of mental health problems is associated with increased risky injection behaviors among PWID. For example, several studies have found an association between depression and syringe sharing [14–19], as well as frequency of injection [14, 20]. A Canadian cohort study of PWID found an association between traumatic life events and depressive symptoms, as well as depressive symptoms and frequency of sharing injecting equipment [21]. Several studies have shown an association between early childhood trauma and an earlier age of first drug use [22–24]. Younger age of onset of IDU is associated with riskier injection behaviors [25]. While most studies of injection risk behavior have occurred in urban settings, a cohort of PWID in rural Appalachia showed rates of RSS comparable to urban-based populations [26].

It is also known that HCV prevalence is higher among people with mental health problems. One case–control study conducted among hospitalized veterans in the 1990s reported that psychiatric conditions were significantly more likely among HCV-infected patients than HCV-negative patients [27]. Among patients hospitalized with mental health problems in Switzerland, HCV prevalence was higher than that of the general population [28]. In North America, pooled data suggest an HCV prevalence of 17.4% among patients with serious mental illness, compared to 1% in the general population [29].

Despite the evidence above separately showing associations between mental health problems and IDU, between mental health problems and risky injection behaviors, and between mental health and HCV, it has not been firmly established whether the association between mental health and HCV infection is mediated by risky injection practices. Furthermore, the majority of existing research on these relationships has been conducted in cohorts of urban PWID, with few studies including PWID that reside in rural areas. Given the increasing vulnerability of rural communities to IDU-associated infections [30], it is particularly important to perform research that informs targeted services in these settings. Using data from a cohort of young adult PWID in rural New Mexico, we sought to examine the association of mental health problems with HCV infection, and to examine whether specific injection practices were mediating factors of this relationship. The results may be used to improve addiction treatment services, as well as related prevention services that are focused on reducing risky drug injection behaviors and HCV incidence among young adults in rural areas.

## Methods

### Study aim

To examine the association between mental health problems and HCV infection in a rural population of young adult PWID. Additionally, we sought to test the following hypothesized theoretical model: mental health problems such as post-traumatic stress disorder (PTSD) may lead to earlier initiation into IDU and riskier injection practices, which may ultimately lead to HCV acquisition.

### Study design, setting and participants

The parent study, named ¡VÁLE!, was a prospective observational study of young adult PWID living in rural areas of New Mexico. The full study protocol is described elsewhere [31]. In brief, the study was conducted in two rural counties of New Mexico. In Rio Arriba County in northern New Mexico, a convenience sample of participants was recruited from September 2016 through May 2018 by screening interested clients of The Mountain Center, a community-based program that hosts a syringe service program as well as other co-located services for PWID. In Doña Ana County in southern New Mexico, participants were recruited from September 2016 through July 2017 by an outreach team in the parking lot of a drop-in center offering harm reduction and health referral services. Eligible participants were 18–29 years old with self-reported IDU in the past 90 days, with no plans of leaving the general area within the following year.

### Study procedures

This secondary analysis utilized cross-sectional data collected at the baseline visit of the ¡VÁLE! Study. The baseline survey included sociodemographic characteristics, exposure risk, drug use history (e.g., age at first drug use), injection behavior (including age first injected) and injecting-related exposures. The injection behavior section asked about risky injection practices, including RSS. The mental health section included questions on prior diagnoses of mental health problems, engagement in mental health services, and medications for mental health conditions. The questionnaire also asked about whether participants desired but did not engage in mental health services and the reasons(s) for lack of engagement. Participants were tested for HCV with an antibody test as well as ribonucleic acid (RNA) test. Participants received a $15 Visa merchandise card for completing the baseline visit.

### Exposures, confounders, mediators, & outcome definitions

#### Exposures

Mental health problems were treated as exposures in the systematic equation modeling (SEM) analysis. Individuals were classified as having any mental health problems if they reported having been diagnosed by a health care provider with at least 1 of the following mental health problems: depression, anxiety, bipolar disorder, borderline personality disorder, schizophrenia, attention deficit disorder/hyperactivity disorder (ADD/ADHD), or PTSD.

#### Confounders

Self-reported data including demographic variables such as age, sex, education, marital status, and race/ethnicity were assessed as potential confounders. We also incorporated educational attainment, insurance status, and history of commercial sex work.

#### Mediators

IDU history and practices were examined as mediators, including: age first injected, duration of IDU, and RSS.

#### History of HCV infection

Participants were classified into two groups. Those who had a positive anti-HCV antibody or positive HCV RNA test were defined as having a history of HCV infection. Those with negative anti-HCV and negative HCV RNA tests were defined as having no history of HCV infection. We did not distinguish between previously infected (antibody positive but RNA negative) and currently infected (i.e. RNA positive) patients in our regression analyses, as we were ultimately interested in their risk of exposure, rather than likelihood of self-clearance or treatment (which would affect their current infection status).

### Statistical analyses

Unless otherwise stated, all analyses were generated using SAS 9.4 software (SAS Institute Inc., Cary, NC, USA). Data are presented as frequencies and percentages for categorical variables and as median and interquartile range (IQR) for continuous variables.

To help identify variables to include in the mediation analyses, we examined the bivariate association between history of HCV infection and the following variables: age first injected, duration of IDU, engagement in RSS, sex at birth, and demographic variables specified above. We also examined bivariate associations between mental health indicators (PTSD diagnosis, any mental health problems), and history of HCV infection, as well as the same set of variables above: age first injected, duration of IDU, engagement in RSS, sex at birth, and other demographic variables. Among mental health problems, PTSD was chosen for separate analysis due to its central role in our theoretical model, in which trauma was the initiating event, and because it was the most prevalent mental health problem reported.

Multivariable modified (robust) Poisson regression (with robust standard errors) was used to estimate the adjusted prevalence ratios (PR) and 95% confidence intervals (95% CI) [32]. For selecting variables to include in the Poisson regression, we chose an approach suggested by Agresti et al., [33]: we started with an a priori list of clinically and epidemiologically important variables. Given the relatively small sample size and discrete distributions of the dependent variables, variables deemed to be significant in the bivariate analyses at a P-value ≤ 0.1 were included in the multivariable modified Poisson regression, as they were suggestive of an effect that warrants further study.

We tested the linearity assumption between each continuous predictor and the dependent variables using cumulative residuals [34]. We also checked multi-collinearity between independent variables. The linearity check indicated that the relationships between duration of IDU and history of HCV infection; between the age first injected and PTSD; and between the age first injected and any mental health problems were not linear (Additional file 1: Tables S3–S5). Therefore, we added the necessary quadratic or cubic terms for duration of IDU or the age first injected in the relevant models (Table 2). The multi-collinearity check showed that the duration of IDU and age first injected were highly correlated and demonstrated collinearity. Therefore, based on model fit, we incorporated only one of the two variables in any Poisson regression models (Table 2).

#### Path analyses

**Fig. 1:**
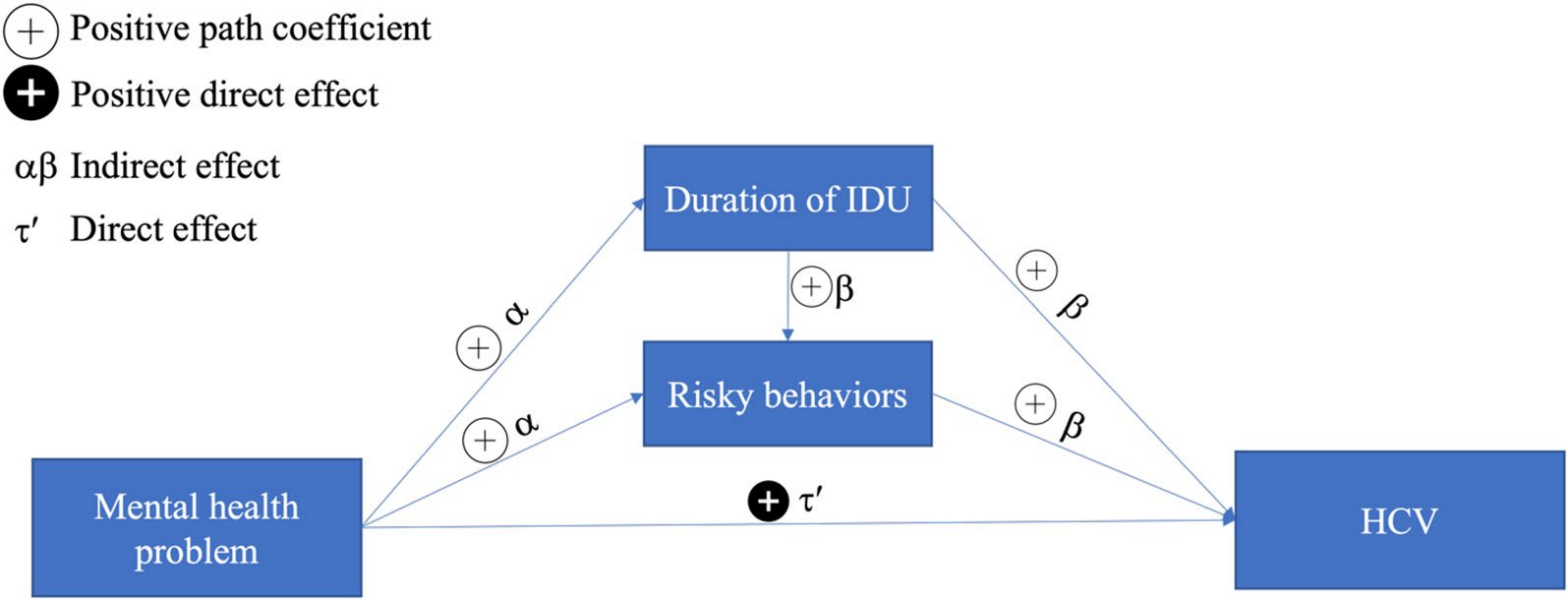
Theoretical model of the mediated association between mental health problems and HCV history. Legend: HCV = Hepatitis C Virus. IDU = Injection Drug Use

An (SEM) framework was used to examine the following proposed theoretical model: Mental health problems→ earlier age first injected/longer duration of IDU→RSS→history of HCV infection (Fig. 1).

$${\text{Legend}}:{\text{ HCV }} = {\text{ Hepatitis C Virus}}.{\text{ IDU }} = {\text{ Injection Drug Use}}$$

We hypothesized that this relationship was confounded by age at enrollment, gender, race/ethnicity, education, marital status, insurance status, and commercial sex work. Therefore, we estimated the total, direct (the path from mental health problems to HCV), and indirect (the path from mental health problems to HCV via the mediators) effects of mental health problems on HCV infection. We used the cross-product method to calculate the indirect effects, which is equal to the product of the coefficient (α) of the path from mental health problems to the mediator, and the coefficient (β) of the path from the mediator to HCV. The total effect (τ) was estimated as the sum of the direct effect coefficient (τ’) and the indirect effect αβ. We first evaluated the theoretical model and then modified it to identify the model that best fits the data. We used coefficient significance, root mean square error of approximation (RMSEA), comparative fit index (CFI), Akaike information criterion (AIC) and Bayesian information criterion (BIC) [35] to identify the model that best fits the data. The SEM analysis was conducted with *M*plus 8.6 [36].

## Results

Baseline data was available for 263 participants. Baseline characteristics are displayed in Table 1 and Additional file 1: Table S1. The median age of participants was 26.1 years (IQR 22.6, 28.2). The majority were Hispanic (87.6%), white (58.8%), single/never married (82.4%), had insurance (91.9%, of which 88.9% were on Medicaid), and had a level of education of high school/GED or above (62.9%). The median age first injected drugs was 19 years (IQR 17.0, 23.0), and slightly more than half of the participants (53.4%) reported engaging in RSS. A minority (1.1%) had been involved in commercial sex work in the last three months. A majority (60.9%) tested positive for HCV antibody, and just under half (45.7%) of all participants tested positive for HCV ribonucleic acid.

**Table 1:** Bivariate associations of history of HCV infection, PTSD, mental health problems, and population characteristics

Variables	Total (N = 263)		History of HCV infection (N = 168)		PTSD (N = 111)		Any mental health problems (N = 158)	
	n (median)	% (IQR)	n (median)	% (IQR)	n (median)	% (IQR)	n (median)	% (IQR)
Age in years	26.1	(22.6–28.2)	**26.9**	**(24.0–28.4)** †	26.7	(22.6–28.2)	26.2	(22.5–28.2)
Age category								
** < **25 years	107	41.0%	**53**	**50.0%** †	44	41.1%	67	62.6%
25 years	154	59.0%	115	75.7%	67	43.5%	91	59.1%
Duration of IDU (years)	4.2	(2.0–8.6)	**5.4**	**(3.3–9.6)** †	**4.9**	**(2.6–8.8)**	4.6	(2.1–8.6)
Age first injected	19.0	(17.0–23.0)	**19.0**	**(17.0–22.0)** †	19.0	(17.0–23.0)	19.0	(17.0–23.0)
Sex at birth								
Male	148	56.7%	101	69.2%	**50**	**33.8%** †	**77**	**52.0%** †
Female	113	43.3%	67	59.8%	61	54.0%	81	71.7%
Hispanic/Latino (a)								
Yes	227	87.6%	146	64.9%	**92**	**40.5%**	**133**	**58.6%** †
No	32	12.4%	21	67.7%	18	56.3%	24	75.0%
Race								
White	151	58.8%	**94**	**63.5%**	66	43.7%	96	63.6%
Black	7	2.7%	2	28.6%	4	57.1%	5	71.4%
Other^a^	99	38.5%	68	68.7%	41	41.4%	55	55.6%
Education								
Less than high school	95	37.1%	62	66.0%	37	39.0%	**52**	**54.7%**
High school/GED and above	161	62.9%	104	64.6%	74	46.0%	106	65.8%
Marital Status								
Single/Never married	211	82.4%	**131**	**62.4%** †	87	41.2%	126	59.7%
Not single	45	17.6%	35	77.8%	24	53.3%	32	71.1%
Insurance/Medicaid								
On Medicaid	200	81.0%	135	67.8%	**86**	**43.0%**	**126**	**63.0%**
On non-Medicaid insurance	27	10.9%	16	59.3%	16	59.3%	19	70.4%
No insurance	20	8.1%	11	55.0%	5	25.0%	9	45.0%
Commercial sex work in last three months								
Yes	3	1.1%	3	100%	2	66.7%	2	66.7%
No	260	98.9%	165	64.7%	109	41.9%	156	60.0%
Positive HCV antibody								
Reactive	156	60.9%	156	100%	73	46.8%	101	64.7%
Non-reactive	100	39.1%	10	10%	37	37.0%	55	55.0%
Positive HCV RNA								
Reactive	105	45.7%	105	100%	45	42.9%	67	63.8%
Non-reactive	125	54.3%	39	31.2%	51	40.8%	74	59.2%
Receptive syringe sharing								
Yes	135	53.4%	**104**	**77.0%** †	56	41.5%	87	64.4%
No	118	46.6%	61	51.7%	54	45.8%	69	58.5%

**Bold text** indicates significance at < 0.10 level, **bold text** with † indicates significance at < 0.05 level

*HCV* Hepatitis C Virus. *IDU* Injection Drug Use. *PTSD* Post-Traumatic Stress Disorder. *RNA* Ribonucleic acid

^a^Other: self-reported as mixed, bi-, or multi-racial, or did not specify

Among the 263 participants, 158 (60%) reported at least one mental health problem. PTSD was the most prevalent condition (42.2%), followed by anxiety (41%), depression (37.3%), and ADD/ADHD (21.3%) (see Additional file 2: Table S2).

In bivariate analyses, a history of HCV infection was associated at p-value < 0.10 with age, duration of IDU, race, marital status, and RSS. PTSD was associated at p-value < 0.10 with duration of IDU, sex, race, and being on non-Medicaid insurance. Having any mental health problems was associated at p-value < 0.10 with female sex, ethnicity, education, and insurance status. (See Table 1).

### Multivariable model

PWID who were 25 years old and above had a higher probability of a history of HCV infection than those who were younger than 25 years old [PR(95% CI) 1.34 (1.08, 1.66) (p < 0.01) (see Table 2). PWID who engaged in RSS had a higher probability of a history of HCV infection [1.33 (1.11, 1.61)] (p < 0.01) than those who did not engage in RSS. PWID who engaged in commercial sex work in the last three months were more likely to have a history of HCV infection than those who did not [1.65 (1.30, 2.10)] (p < 0.01), although the sample of those reporting commercial sex work was only 3 individuals. Female PWID were more likely to have PTSD [1.62 (1.21, 2.17)] (p = 0.001) and any mental health problems [1.43 (1.18, 1.73)] (p < 0.001) compared to male PWID. Non-Hispanic PWID were more likely than Hispanic PWID to have PTSD [1.33 (0.95, 1.85)] (p < 0.10) and any mental health problems [1.25 (1.01, 1.54)] (p = 0.04). PWID who were on non-Medicaid insurance were more likely to have PTSD [1.47 (1.06, 2.03)] (p = 0.02) than those who were on Medicaid insurance, although the sample of non-Medicaid insurance was considerably smaller (27), than those on Medicaid insurance (200). PWID who had a history of HCV infection were more likely to have PTSD than those who did not have a history of HCV infection [1.42 (1.03, 1.96)] (p = 0.03). PWID with less than a high school level of education were more likely to have any mental health problems than those with high school education/GED or above [1.22 (0.99, 1.50)] (p = 0.07).

**Table 2:** Adjusted modified (robust) Poisson regression models for history of HCV infection, PTSD, and any mental health problems

Variables	HCV infection		PTSD		Any mental health problems	
	Relative Risk	P-value	Relative Risk	P-value	Relative Risk	P-value
Sex at birth						
Male			Ref		Ref	
Female			1.62 (1.21, 2.17)	< 0.01	1.43 (1.18, 1.73)	< 0.01
Age category						
> = 25 years	1.34 (1.08, 1.66)	< 0.01				
< 25 years	Ref					
Duration of IDU	1.35 (1.10, 1.66)	< 0.01				
Duration of IDU quadratic	0.97 (0.94, 0.99)	0.02				
Duration of IDU cubic	1.00 (1.00, 1.00)	0.02				
Age first injected			0.76 (0.64, 0.89)	< 0.01	0.83 (0.73, 0.95)	< 0.01
Age first injected quadratic			1.01 (1.00, 1.01)	< 0.01	1.00 (1.00, 1.01)	0.02
Receptive syringe sharing						
Yes	1.33 (1.11, 1.61)	< 0.01	0.80 (0.60, 1.06)	0.12	1.04 (0.85, 1.27)	0.74
No	Ref		Ref		Ref	
Hispanic/Latino (a)						
Yes			Ref		Ref	
No			1.33 (0.95, 1.85)	0.10	1.25 (1.01, 1.54)	0.04
Marital Status						
Single/Never Married	Ref					
Not Single	1.11 (0.92, 1.34)	0.26				
Insurance/Medicaid						
On Medicaid			Ref			
On non-Medicaid Insurance			1.47 (1.06, 2.03)	0.02		
Not on insurance			0.67 (0.31, 1.46)	0.32		
Commercial sex work in last 3 months						
Yes	1.65 (1.30, 2.10)	< 0.01				
No	Ref					
History of HCV infection						
Yes			1.42 (1.03, 1.96)	0.03	1.16 (0.93, 1.45)	0.18
No			Ref		Ref	
Education						
Less than high school					1.22 (0.99, 1.50)	0.07
High school/GED and above					Ref	

Any variables that were hypothesized to be associated with the dependent variable, in addition to any variables with p < 0.10 significance, were included in the modified robust Poisson simple regression model. A blank space indicates that the variable was not used in the regression model for that dependent variable

*GED* General educational development. *HCV* Hepatitis C Virus. *IDU* Injection drug use. *PTSD* Post-Traumatic stress disorder

We detected a significant non-linear association between age first injected and PTSD, age first injected and having any mental health problem, as well as between duration of IDU and HCV infection. Specifically, there were convex relationships between age first injected and PTSD, and between age first injected and having any mental health problems (models included quadratic term). On the other hand, there was a concave relationship between duration of IDU and HCV infection (quadratic term). There was a positive cubic trend, indicating that the quadratic trend is increasingly positive with the increase of duration of IDU. For all outcomes, results of the models that include (i) the linear terms only, (ii) the quadratic terms, and (iii) the cubic terms are shown in the Additional file 2: Table S3, Additional file 3: Table S4, and Additional file 4: Table S5, respectively.

### Path analyses

#### Association between PTSD and history of HCV infection

**Fig. 2:**
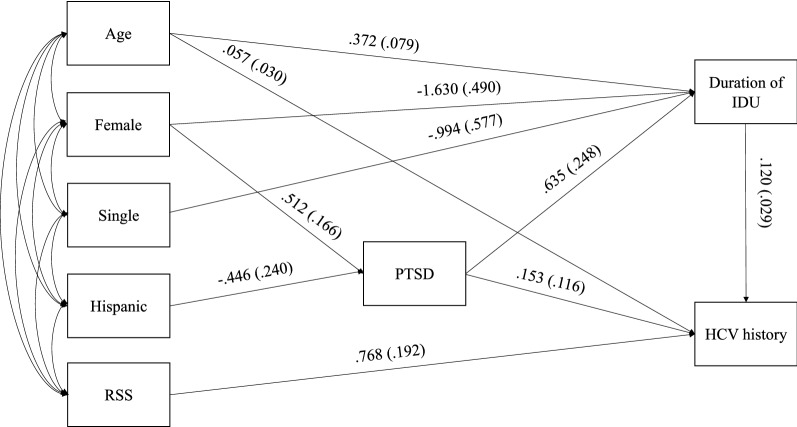
Path analysis, direct and indirect effects of PTSD on HCV history. Legend: HCV = Hepatitis C Virus. IDU = Injection Drug Use. PTSD = Post-Traumatic Stress Disorder. RSS = Receptive Syringe Sharing

The model had a good fit (CFI = 0.93, RMSEA (90% CI) 0.057 (0.0,0.102), SRMR = 0.077). Adjusting for covariates, participants with longer duration of IDU (β = 0.120, P < 0.01) were more likely to have a history of HCV infection. In addition, PTSD (α = 0.635, P = 0.01), age (β = 0.372, P < 0.01), and female sex (β = -1.630, P =  < 0.01) were significantly associated with duration of IDU. Hispanic participants were less likely to report PTSD than non-Hispanic participants (β = -0.446, P = 0.06), and females were more likely to report PTSD than males (β = 0.512, P =  < 0.01). We identified a significant indirect effect (αβ = 0.077 P = 0.03) between PTSD and history of HCV infection. More specifically, earlier initiation of IDU mediated the effect of PTSD on HCV history. Adjusting for the mediator, the direct path from PTSD to HCV was not significant (τ’ = 0.153, P = 0.19) (Table 3, Fig. 2). RSS was not associated with PTSD; therefore, the path between PTSD and RSS was not retained in the final model.

**Table 3:** Unstandardized results of HCV on PTSD mediation analysis

	Estimate	S.E	Est./S.E	P-Value (Two-Tailed)
History of HCV infection regressed on				
Age in years	0.057	0.030	1.894	0.06
Duration of IDU	0.120	0.029	4.186	< 0.01
Receptive syringe sharing	0.625	0.140	4.472	< 0.01
PTSD	0.153	0.116	1.324	0.19
Duration of IDU regressed on				
PTSD	0.635	0.248	2.559	0.01
Age in years	0.372	0.079	4.712	< 0.01
Female	− 1.630	0.490	− 3.323	< 0.01
Single	− 0.994	0.577	− 1.724	0.09
PTSD regressed on				
Female	0.512	0.166	3.079	< 0.01
Hispanic/Latino	− 0.446	0.240	− 1.857	0.06
Effects from PTSD to duration of IDU to HCV history				
Total	0.230	0.118	1.943	0.05
Indirect	0.077	0.035	2.197	0.03
Direct	0.153	0.116	1.324	0.19

*HCV* Hepatitis C Virus. *IDU* Injection Drug Use. *PTSD* Post-Traumatic Stress Disorder. *S.E* Standard Error

#### Association between any mental health problems and history of HCV infection

**Fig. 3:**
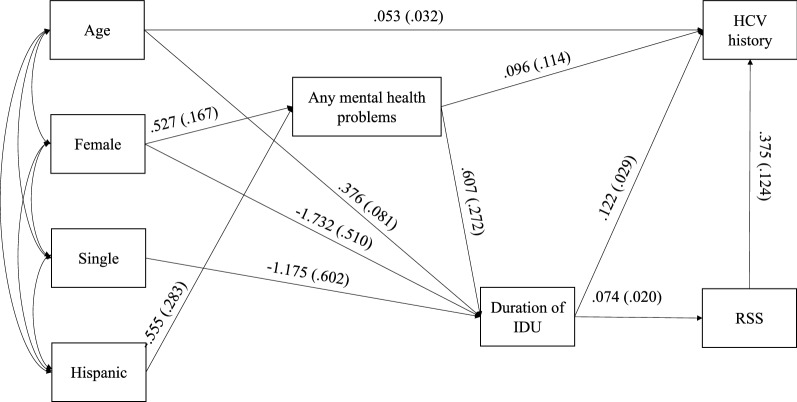
Path analysis, direct and indirect effects of mental health problems on HCV history. Legend: HCV = Hepatitis C Virus. IDU = Injection Drug Use. RSS = Receptive Syringe Sharing

The model depicting the association between any mental health problems and history of HCV infection had an excellent fit (CFI = 1, RMSEA (90% CI) 0 (0,0.021), SRMR = 0.052). Results were in the same direction observed for the PTSD/history of HCV infection model, although the total effect of having any mental health problems was not significantly associated with HCV (τ = 0.19, P = 0.12). There was, however, a significant indirect relationship between any mental health problems and HCV, such that the association was partially mediated by duration of IDU and the sequential mediation of duration of IDU and RSS (total indirect effect = αβ + αββ = 0.091, P = 0.05). (Table 4, Fig. 3).

**Table 4:** Unstandardized results of HCV on any mental health problems mediation analysis

	Estimate	S.E	Est./S.E	P-value (Two-Tailed)
History of HCV infection regressed on				
Age in years	0.053	0.032	1.649	0.10
Receptive syringe sharing	0.375	0.124	3.021	< 0.01
Duration of IDU	0.122	0.029	4.178	< 0.01
Any mental health problems	0.096	0.114	0.842	0.40
Duration of IDU regressed on				
Any mental health problems	0.607	0.272	2.232	0.03
Age in years	0.376	0.081	4.643	< 0.01
Female	− 1.732	0.510	− 3.395	< 0.01
Single	− 1.175	0.602	− 1.950	0.05
Receptive syringe sharing regressed on				
Duration of IDU	0.272	0.069	3.917	< 0.01
Any mental health problems regressed on				
Female	0.527	0.167	3.147	< 0.01
Hispanic/Latino	− 0.555	0.283	− 1.961	0.05
Effects from any mental health problems to duration of IDU/RSS to HCV history				
Total	0.187	0.119	1.573	0.12
Total indirect	0.091	0.045	2.002	0.05
Indirect via duration of IDU	0.074	0.038	1.963	0.05
Indirect via duration of IDU-> RSS	0.017	0.011	1.531	0.13
Direct	0.096	0.114	0.842	0.40

*HCV* Hepatitis C Virus. *IDU* Injection Drug Use. *S.E*. Standard Error

## Discussion

In a cohort of young PWID living in rural areas of New Mexico, a self-reported history of PTSD was significantly associated with history of HCV infection, and this relationship was partially mediated by duration of IDU. We observed an indirect association between having any mental health problems and HCV infection, which was partially mediated by duration of IDU. While RSS itself was significantly associated with HCV infection, it did not appear to serve as a mediator of the relationship between PTSD and HCV infection. The association between mental health problems and HCV infection was partially mediated by the sequential mediation of duration of IDU and RSS. In this cohort, we found a high prevalence of mental health problems (60%), and PTSD (42%) in particular.

Our study adds to the body of literature that has shown that mental health problems are associated with risky injection practices. [14, 18, 21, 22]. Our study goes a step further in delineating the nuances of these relationships using SEM. Our theoretical model was partially supported by our SEM analysis; the relationship between mental health problems, specifically PTSD, and HCV infection was at least partially mediated by duration of IDU and sequential mediation of duration of IDU and RSS.

The prevalence of PTSD in this cohort is consistent with the 30% to 59% prevalence reported in PWID or people with SUDs in other studies, with higher rates among females [37–39]. Many of these studies were completed in urban areas. Few prior studies have investigated the impact of rurality on PTSD in PWID, although research suggests that individuals with opioid use disorder in rural areas may have higher rates of exposure to traumatic events in childhood [13]. In our cohort of PWID in rural New Mexico, PTSD rates were comparable to similar populations in urban settings.

There were some interesting and unexpected findings. For example, our analysis found that females were more likely to report PTSD, but females had an older age of onset of IDU. Previous studies examining sex differences in mental health among PWID have reported mixed findings. National data suggests that women with SUDs are more likely to have co-occurring mood disorders [40]. A cohort in Canada found that female PWID were more likely to have mental health problems at study entry and they were also more likely to be diagnosed with mental health problems during follow-up [41]. In one Canadian cohort examining injecting behaviors and psychological distress, the relationship between psychological distress, binge drug injection, and sharing injection equipment was weaker among women than among men [42, 43]. However, in another cohort, females were more likely to share needles, sharers reported higher levels of depression than non-sharers, and female sharers reported the highest levels of depression in all groups [44].

Our study also found that individuals who identified as Hispanic (the majority of the sample) were less likely to report a history of PTSD or other mental health problems, although the confidence in this conclusion is limited by the small sample size of non-Hispanic individuals in our cohort. One possible explanation is underdiagnosis: it has been posited that racial/ethnic differences between clinicians and clients may contribute to clinician difficulties in accurate assessment and diagnosis of client’s symptoms [45]. Alternatively, there may also be true race/ethnicity differences in rates of mental health problems. Investigators have examined this phenomenon, with some studies suggesting lower lifetime prevalence of mental disorders in Black, Latino, and Asian individuals, compared to White individuals. However, this effect may be largely mediated by whether the individual or their parent is foreign-born (not assessed in our study), as foreign-born individuals have been found to have lower rates of lifetime mental disorders, the so-called “immigrant paradox”[46, 47]. A final explanation could be differential item functioning, in which Hispanic and non-Hispanic individuals understood the questionnaire differently. Investigators have examined the complex interplay of these factors in PTSD assessment by race/ethnicity [48]. Finally, it is worth noting that there is substantial heterogeneity among characteristics of people in the US who identify as Hispanic or Latino, and studies have found different rates of mental health and SUDs for Mexican, Cuban, and Puerto-Rican individuals [49].

Notably, a high proportion of our participants (91.9%) had insurance, and 76% of the total sample was on Medicaid. This is a similar proportion to that seen in analysis of healthcare insurance status among PWID in urban settings across the USA in 2018, which found that in Medicaid expansion states, 71% had Medicaid coverage (vs 14% in non-Medicaid expansion states) [50]. New Mexico implemented expanded Medicaid in 2014, before our study period, and it is encouraging to see the reach of this expanded access to rural counties. An analysis of naloxone access disparities in Southeast Michigan (another Medicaid expansion state) found similar high levels of insurance (91–92%) in both urban and rural/suburban settings [51].

Our study has several important implications for addiction treatment and HCV prevention services for young adult PWID in rural settings. Given the complex interplay between mental health (particularly PTSD), early injection initiation, risky injection practices, and HCV acquisition, services that focus on improving the well-being of PWID may benefit from an integrated, multidisciplinary approach. Harm reduction programs (such as syringe service programs), behavioral health services, and infectious disease providers are likely to have improved efficacy if they act in concert, screening for and addressing multiple interacting comorbidities. Further, given the high prevalence of PTSD and mental health problems among this young cohort and its association with earlier injection initiation, this cascade of risk may be initiated by adverse childhood events, as has been suggested by several studies in the literature [52, 53]. Screening and intervention for adverse childhood events early in life may be beneficial, and treatment services should be appropriate for young people with SUDs, who often have lower rates of treatment [54]. Early identification of traumatic events and mental health problems among young adult PWID should be expanded in rural addiction treatment programs, general medical settings, and community-based harm reduction programs. Given that many rural communities have been identified as high risk for HCV outbreaks [5], and have also been identified as healthcare shortage areas [55], federal assistance may be needed to facilitate implementation of these services into existing addiction treatment programs in rural areas.

Our study has some methodological limitations. First, we rely on self-reported data, which is subject to potential recall and reporting bias. Second, the temporality of relationships cannot be fully extrapolated from our cross-sectional data. For example, our hypothesis is that trauma may have preceded PTSD, which may have preceded IDU and HCV acquisition. But it is also possible that IDU began prior to experiencing trauma and PTSD, and it is also possible that HCV acquisition preceded any of the other factors. Lastly, our data does not include information on the severity of mental health. Some studies among PWID have used depression scales and other instruments to measure severity, while our survey asked only about a history of being diagnosed with a condition. Additionally, prospective studies are needed to determine whether improved integration of mental health care and/or trauma-informed services reduces risky injection drug practices and HCV incidence among young PWID in rural areas.

## Conclusions

In a cohort of young rural PWID in New Mexico, we found that PTSD is associated with a history of HCV infection, and that this relationship is mediated by duration of IDU. The relationship between any mental health problems and HCV infection is mediated by duration of IDU, as well as sequential mediation of duration of IDU and RSS. Prevention of risky injection practices and integrated trauma-informed mental health care may be critical components of addiction treatment in rural areas and may inform broader community efforts to reduce HCV incidence in rural communities.

## Supplementary Information

**Additional file 1: Table S1.** Baseline population characteristics. **Table S2.** Mental health problems and engagement in care.

**Additional file 2: Table S3.** Adjusted modified (robust) Poisson regression models for history of HCV infection, PTSD, and any mental health problems

**Additional file 3: Table S4.** Adjusted modified (robust) Poisson regression models for history of HCV infection, PTSD, and any mental health problems.

**Additional file 4: Table S5.** Adjusted modified (robust) Poisson regression models for history of HCV infection, PTSD, and any mental health problems.

## Data Availability

The datasets generated and/or analyzed during the current study are not publicly available due to lack of data sharing acknowledgement of de-identified data in the consent and IRB protocol. The request for data sharing can be considered on a case-by-case basis with a formal data sharing agreement between institutions.

## Abbreviations

ADD/ADHD: Attention deficit disorder/hyperactivity disorder
AIC: Akaike information criterion BIC—bayesian information criterion
CFI: Comparative fit index
CI: Confidence intervals
HCV: Hepatitis C virus
IDU: Injection drug use
IQR: Interquartile range
PR: Prevalence ratio
PTSD: Post-traumatic stress disorder
PWID: People who inject drugs
RMSEA: Root mean square error of approximation
RNA: Ribonucleic acid
RSS: Receptive syringe sharing
SEM: Structural equation modeling
SUD: Substance use disorder
