# Supplementary material for: Association of co-occurring mental health problems with hepatitis C status among young people who inject drugs in rural New Mexico, 2016–2018
Source: Addict Sci Clin Pract. 2022 Oct 20;17:58. doi: 10.1186/s13722-022-00340-3 (PMC9583516; doi:10.1186/s13722-022-00340-3)
Supplement: Supplementary file 1 — Additional file 1: Table S1. Baseline population characteristics. Table S2. Mental health problems and engagement in care. [file 13722_2022_340_MOESM1_ESM.docx]

| **Supplemental Table 1**. Baseline population characteristics | | | | |
| --- | --- | --- | --- | --- |
| Characteristic | N | N missing | n or Mean | % or SD |
| Age in years | 261 | 2 | 25.38 | 3.26 |
| Duration of IDU (years) | 254 | 9 | 5.32 | 3.85 |
| Age first injected | 254 | 9 | 19.99 | 4.07 |
| Female sex at birth | 261 | 2 | 113 | 43.3% |
| Hispanic/Latino(a) | 259 | 4 | 227 | 87.6% |
| Finished high school diploma/GED or greater | 256 | 7 | 161 | 62.9% |
| Single/Never Married | 256 | 7 | 211 | 82.4% |
| Race/Ethnicity: |  |  |  |  |
| White/Caucasian/European American | 263 | 0 | 151 | 57.4% |
| Black/African-American | 263 | 0 | 7 | 2.7% |
| Asian/Asian-American | 263 | 0 | 0 | 0% |
| Filipino/a or Pacific Islander | 263 | 0 | 1 | 0.4% |
| Native American | 263 | 0 | 22 | 8.4% |
| Other | 263 | 0 | 84 | 31.9% |
| Has health insurance coverage | 255 | 8 | 227 | 91.9% |
| Medicaid | 225 | 38 | 200 | 88.9% |
| Commercial Sex Work in last 3 months | 263 | 0 | 3 | 1.14% |
| Baseline HCV testing |  |  |  |  |
| Positive HCV antibody | 256 | 7 | 156 | 60.9% |
| Positive HCV RNA | 230 | 33 | 105 | 45.7% |
| Receptive syringe sharing | 253 | 10 | 135 | 53.4% |

GED = General Educational Development. HCV = Hepatitis C Virus. IDU = Injection Drug Use. RNA - ribonucleic acid. SD = Standard Deviation.

| **Supplemental table 2**. Mental health problems and engagement in care |  |  |  |  |
| --- | --- | --- | --- | --- |
| Characteristic | N | N missing | n | % |
| Mental health counseling, therapy, or treatment in the last three months? | 254 | 9 | 54 | 21.3% |
| Type of mental health provider |  |  |  |  |
| Counselor, SW, Psychologist or therapist | 54 | 0 | 33 | 61.1% |
| Psychiatrist | 54 | 0 | 17 | 31.5% |
| PCP | 54 | 0 | 1 | 1.9% |
| Other | 54 | 0 | 3 | 5.6% |
| Spoken to any provider about mental health counseling | 188 | 12 | 48 | 25.5% |
| Ever been diagnosed with: |  |  |  |  |
| Depression | 263 | 0 | 98 | 37.3% |
| Anxiety | 263 | 0 | 108 | 41.1% |
| Bipolar Disorder | 263 | 0 | 39 | 14.8% |
| Borderline Personality Disorder | 263 | 0 | 4 | 1.5% |
| Schizophrenia | 263 | 0 | 9 | 3.4% |
| ADD/ADHD | 263 | 0 | 56 | 21.3% |
| PTSD | 263 | 0 | 111 | 42.2% |
| Other | 263 | 0 | 14 | 5.3% |
| Don't Know/Unsure of diagnosis | 263 | 0 | 2 | 0.8% |
| Any of the above | 263 | 0 | 158 | 60.1% |
| Never been diagnosed with a psychiatric diagnosis | 263 | 0 | 92 | 35.0% |
| Taking medication for: |  |  |  |  |
| Depression | 97 | 1 | 18 | 18.6% |
| Anxiety | 108 | 0 | 34 | 31.5% |
| Bipolar Disorder | 39 | 0 | 8 | 20.5% |
| Borderline Personality Disorder | 3 | 1 | 1 | 33.3% |
| Schizophrenia | 9 | 0 | 2 | 22.2% |
| ADD/ADHD | 55 | 1 | 2 | 3.6% |
| PTSD | 111 | 0 | 23 | 20.7% |
| Other diagnoses | 14 | 0 | 5 | 35.7% |
| Taking psychiatric medication for at least one of the above mental health conditions | 263 | 0 | 45 | 17.1% |
| Wanted to see someone for mental health reasons and not gone, last three months? | 247 | 16 | 56 | 22.7% |
| Reasons for not having seen someone for mental health care, last three months: |  |  |  |  |
| Worried seeing a provider won't help | 56 | 0 | 9 | 16.1% |
| Do not know where to go | 56 | 0 | 16 | 28.6% |
| Do not feel well enough to go (sick, tired, weak, sad) | 56 | 0 | 15 | 26.8% |
| Cannot get into a program/provider - waitlist or not taking new clients | 56 | 0 | 12 | 21.4% |
| Cannot afford it | 56 | 0 | 7 | 12.5% |
| Do not like programs/providers available, do not fit needs | 56 | 0 | 6 | 10.7% |
| Transportation is difficult | 56 | 0 | 11 | 19.6% |
| Getting the free time to go is difficult | 56 | 0 | 12 | 21.4% |
| Afraid of being judged or treated badly by family, friends, or others in the community who find out | 56 | 0 | 3 | 5.4% |
| Afraid of being judged or treated badly by the treatment staff | 56 | 0 | 3 | 5.4% |
| Worried treatment will be unpleasant and interfere with life | 56 | 0 | 3 | 5.4% |
| Actively drinking and using drugs | 56 | 0 | 13 | 23.2% |

ADD/ADHD = Attention Deficit Disorder/Hyperactivity Disorder. PCP = Primary Care Provider. PTSD = Post-Traumatic Stress Disorder. SW = Social Worker.
