# Supplementary material for: Association of co-occurring mental health problems with hepatitis C status among young people who inject drugs in rural New Mexico, 2016–2018
Source: Addict Sci Clin Pract. 2022 Oct 20;17:58. doi: 10.1186/s13722-022-00340-3 (PMC9583516; doi:10.1186/s13722-022-00340-3)
Supplement: Supplementary file 2 — Additional file 2: Table S3. Adjusted modified (robust) Poisson regression models for history of HCV infection, PTSD, and any mental health problems [file 13722_2022_340_MOESM2_ESM.docx]

| **Supplemental table 3 (linear terms only)**. Adjusted modified (robust) Poisson regression models for history of HCV infection, PTSD, and any mental health problems | | | | | | | | | |
| --- | --- | --- | --- | --- | --- | --- | --- | --- | --- |
| Variables | HCV infection | | | PTSD | | | Any mental health problems | | |
|  | Relative Risk | P-value | Relative Risk | | P-value | Relative Risk | | P-value |  |
| Sex at birth |  |  |  | |  |  | |  |  |
| Male |  |  | Ref | |  | Ref | |  |  |
| Female |  |  | 1.58 (1.19, 2.11) | | <0.01 | 1.41 (1.16, 1.71) | | <0.01 |  |
|  |  |  |  | |  |  | |  |  |
| Age category |  |  |  | |  |  | |  |  |
| >= 25 years | 1.32 (1.06, 1.65) | 0.01 |  | |  |  | |  |  |
| < 25 years | Ref |  |  | |  |  | |  |  |
|  |  |  |  | |  |  | |  |  |
| Duration of IDU | 1.05 (1.03, 1.07) | <0.01 |  | |  |  | |  |  |
|  |  |  |  | |  |  | |  |  |
| Age first injected |  |  | 0.98 (0.94, 1.02) | | 0.25 | 0.98 (0.96, 1.00) | | 0.08 |  |
|  |  |  |  | |  |  | |  |  |
| Receptive syringe sharing |  |  |  | |  |  | |  |  |
| Yes | 1.36 (1.13, 1.65) | <0.01 | 0.80 (0.60, 1.06) | | 0.12 | 1.03 (0.84, 1.26) | | 0.76 |  |
| No | Ref |  | Ref | |  | Ref | |  |  |
|  |  |  |  | |  |  | |  |  |
| Hispanic/Latino(a) |  |  |  | |  |  | |  |  |
| Yes |  |  | Ref | |  | Ref | |  |  |
| No |  |  | 1.33 (0.94, 1.89) | | 0.10 | 1.25 (1.01, 1.55) | | 0.04 |  |
|  |  |  |  | |  |  | |  |  |
| Marital status |  |  |  | |  |  | |  |  |
| Single/Never married | Ref |  |  | |  |  | |  |  |
| Not single | 1.09 (0.91, 1.31) | 0.34 |  | |  |  | |  |  |
|  |  |  |  | |  |  | |  |  |
| Insurance/Medicaid |  |  |  | |  |  | |  |  |
| Medicaid |  |  | Ref | |  |  | |  |  |
| **N**on-Medicaid Insurance |  |  | 1.45 (1.04, 2.03) | | 0.03 |  | |  |  |
| Not on insurance |  |  | 0.68 (0.31, 1.46) | | 0.32 |  | |  |  |
|  |  |  |  | |  |  | |  |  |
| Commercial sex work**,** last 3 months |  |  |  | |  |  | |  |  |
| Yes | 1.75 (1.28, 2.38) | <0.01 |  | |  |  | |  |  |
| No |  |  |  | |  |  | |  |  |
|  |  |  |  | |  |  | |  |  |
| History of HCV infection |  |  |  | |  |  | |  |  |
| Yes |  |  | 1.41 (1.02, 1.95) | | 0.04 | 1.17 (0.93, 1.46) | | 0.17 |  |
| No |  |  | Ref | |  | Ref | |  |  |
|  |  |  |  | |  |  | |  |  |
| Education |  |  |  | |  |  | |  |  |
| Less than high school |  |  |  | |  | 1.21 (0.98, 1.49) | | 0.07 |  |
| High school/GED and above |  |  |  | |  | Ref | |  |  |

Any variables that were hypothesized to be associated with the dependent variable, in addition to any variables with p < 0.10 significance, were included in the modified robust Poisson simple regression model.

A blank space indicates that the variable was not used in the regression model for that dependent variable.
