# Supplementary material for: Association of co-occurring mental health problems with hepatitis C status among young people who inject drugs in rural New Mexico, 2016–2018
Source: Addict Sci Clin Pract. 2022 Oct 20;17:58. doi: 10.1186/s13722-022-00340-3 (PMC9583516; doi:10.1186/s13722-022-00340-3)
Supplement: Supplementary file 3 — Additional file 3: Table S4. Adjusted modified (robust) Poisson regression models for history of HCV infection, PTSD, and any mental health problems. [file 13722_2022_340_MOESM3_ESM.docx]

| Supplemental table 4 (quadratic terms). Adjusted modified (robust) Poisson regression models for history of HCV infection, PTSD, and any mental health problems | | | | | | |
| --- | --- | --- | --- | --- | --- | --- |
| Variables | HCV infection | | PTSD | | Any mental health problems | |
|  | Relative Risk | P-value | Relative Risk | P-value | Relative Risk | P-value |
| Sex at birth |  |  |  |  |  |  |
| Male |  |  | Ref |  | Ref |  |
| Female |  |  | 1.62 (1.21, 2.17) | <0.01 | 1.43 (1.18, 1.73) | <0.01 |
|  |  |  |  |  |  |  |
| Age category |  |  |  |  |  |  |
| >= 25 years | 1.32 (1.07, 1.64) | 0.01 |  |  |  |  |
| < 25 years | Ref |  |  |  |  |  |
|  |  |  |  |  |  |  |
| Duration of IDU | 1.14 (1.05, 1.23) | <0.01 |  |  |  |  |
|  |  |  |  |  |  |  |
| Duration of IDU quadratic | 0.99 (0.99, 1.00) | 0.01 |  |  |  |  |
|  |  |  |  |  |  |  |
| Age first injected |  |  | 0.76 (0.64, 0.89) | <0.01 | 0.83 (0.73, 0.95) | <0.01 |
|  |  |  |  |  |  |  |
| Age first injected quadratic |  |  | 1.01 (1.00, 1.01) | <0.01 | 1.00 (1.00, 1.01) | 0.02 |
|  |  |  |  |  |  |  |
| Receptive syringe sharing |  |  |  |  |  |  |
| Yes | 1.34 (1.11, 1.62) | <0.01 | 0.80 (0.60, 1.06) | 0.12 | 1.04 (0.85, 1.27) | 0.74 |
| No | Ref |  | Ref |  | Ref |  |
|  |  |  |  |  |  |  |
| Hispanic/Latino(a) |  |  |  |  |  |  |
| Yes |  |  | Ref |  | Ref |  |
| No |  |  | 1.33 (0.95, 1.85) | 0.10 | 1.25 (1.01, 1.54) | 0.04 |
|  |  |  |  |  |  |  |
| Marital status |  |  |  |  |  |  |
| Single/Never married | Ref |  |  |  |  |  |
| Not single | 1.08 (0.90, 1.30) | <0.41 |  |  |  |  |
|  |  |  |  |  |  |  |
| Insurance/Medicaid |  |  |  |  |  |  |
| On Medicaid |  |  | Ref |  |  |  |
| On non-Medicaid Insurance |  |  | 1.47 (1.06, 2.03) | 0.02 |  |  |
| Not on insurance |  |  | 0.67 (0.31, 1.46) | 0.32 |  |  |
|  |  |  |  |  |  |  |
| Commercial sex work in last 3 months |  |  |  |  |  |  |
| Yes | 1.66 (1.23, 2.23) | <0.01 |  |  |  |  |
| No | Ref |  |  |  |  |  |
|  |  |  |  |  |  |  |
| History of HCV infection |  |  |  |  |  |  |
| Yes |  |  | 1.42 (1.03, 1.96) | 0.03 | 1.16 (0.93, 1.45) | 0.18 |
| No |  |  | Ref |  | Ref |  |
|  |  |  |  |  |  |  |
| Education |  |  |  |  |  |  |
| Less than high school |  |  |  |  | 1.22 (0.99, 1.50) | 0.07 |
| High school/GED and above |  |  |  |  | Ref |  |

Any variables that were hypothesized to be associated with the dependent variable, in addition to any variables with p < 0.10 significance, were included in the modified robust Poisson simple regression model.

A blank space indicates that the variable was not used in the regression model for that dependent variable.
